# Supplementary material for: Hepatocytes trap and silence coxsackieviruses, protecting against systemic disease in mice
Source: Commun Biol. 2020 Oct 16;3:580. doi: 10.1038/s42003-020-01303-7 (PMC7568585; doi:10.1038/s42003-020-01303-7)
Supplement: Supplementary file 1 — Supplementary Information [file 42003_2020_1303_MOESM1_ESM.pdf]

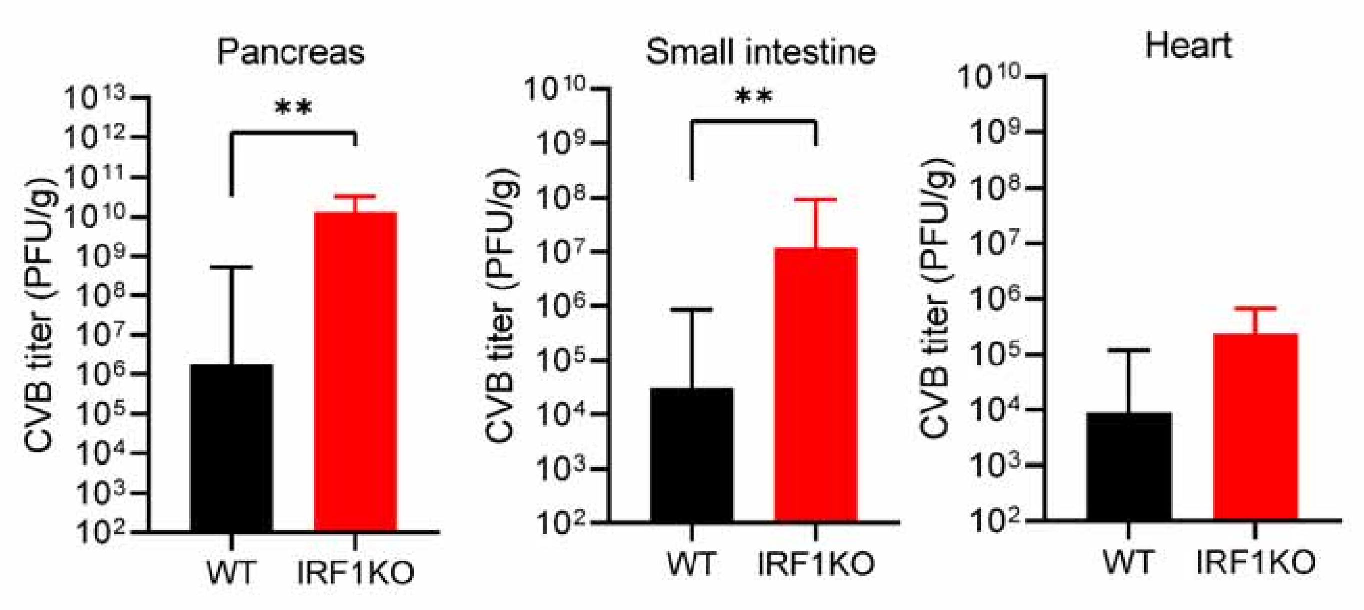

Supplementary Fig. 1. At 24 hours post infection, CVB3 titers are elevated in IRF1KO mice

WT and IRF1KO mice were infected with 10^4 pfu of CVB3 (i.p.), and were sacrificed 24 hours later. CVB3 titers in the indicated tissues were determined by standard plaque assay. P values less than 0.05 were considered significant, and are indicated in figures as follows: * $0.05>p>0.01$; ** $0.01 \geq p>0.001$; *** $0.001 \geq p>0.0001$; **** $p \leq 0.0001$.

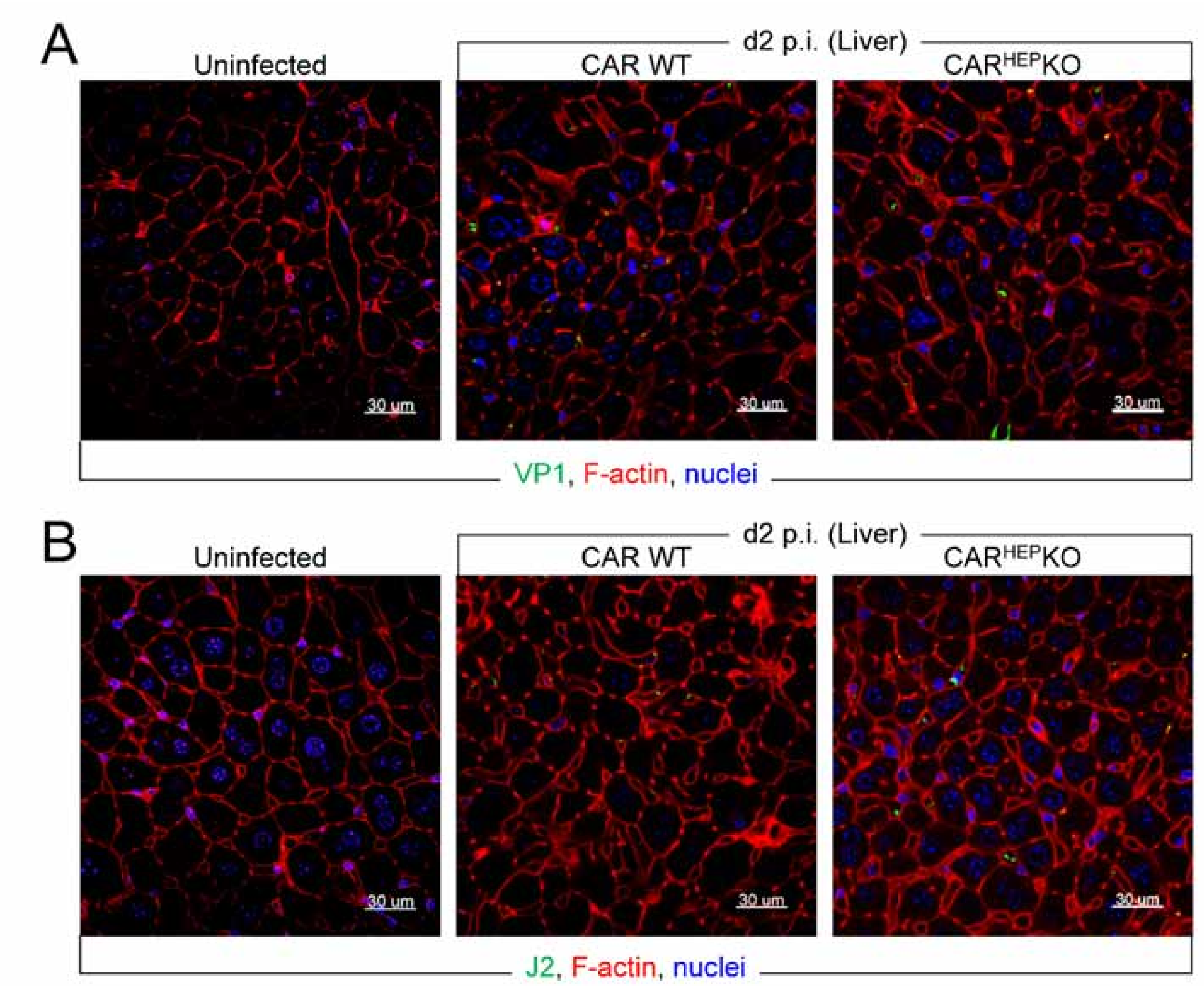

Supplementary Fig. 2. At d2 p.i., CVB3 VP1 and dsRNA signals are detected almost solely in the sinusoids of both WT and CAR^{HEP}KO mice, and are present at similar frequency in both mouse strains.

Immunohistochemistry of the livers from CAR WT and CAR^{HEP}KO mice at 2 days after CVB3 infection (10^4 pfu) using antibody against VP1 (green, panel A) or against dsRNA (J2 antibody,green, panel B). Blue; Nuclei, Red; F-Actin. Green signals are observed exclusively in sinusoids.

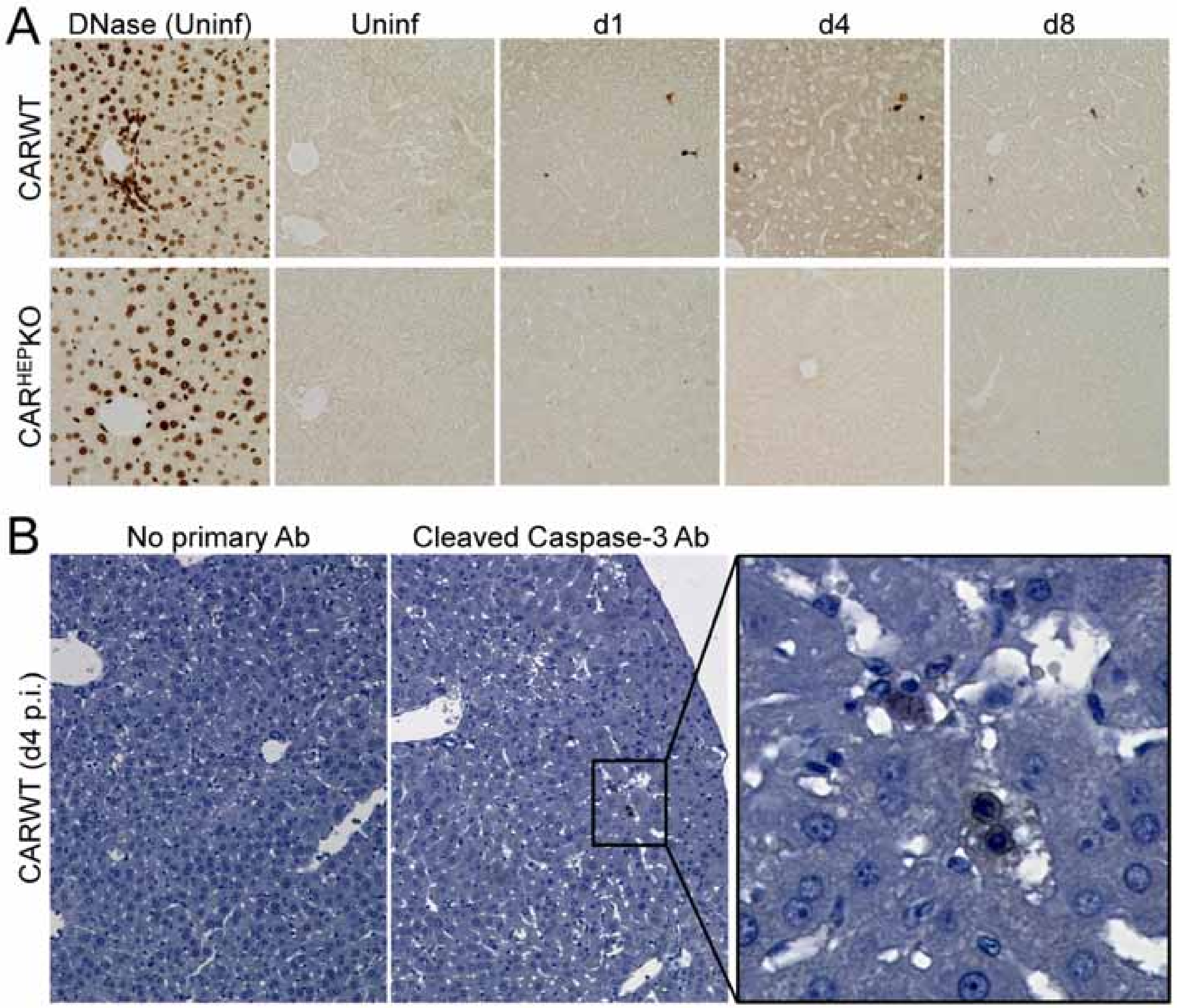

Supplementary Fig. 3. CVB3 infection causes limited hepatic apoptosis, which is dependent on CAR expression by hepatocytes and is not accompanied by inflammation.

Panel A. WT and $CAR^{HEP}KO$ mice were infected with CVB3 (10^4 pfu, i.p.) and were sacrificed at the indicated times post infection. Liver sections were subjected to a TUNEL assay using a commercially available TUNEL Assay kit-HRP-DAB (abcam, #ab206.386), according to the manufacturer's instructions. DNase-treated samples of uninfected tissues were used as the assay control. Panel B. Sections of liver from WT mice at d4 p.i. were stained for cleaved caspase 3, another marker of apoptotic cells. Such cells were infrequent and, as shown by the enlarged inset, were not accompanied by significant inflammatory infiltration.

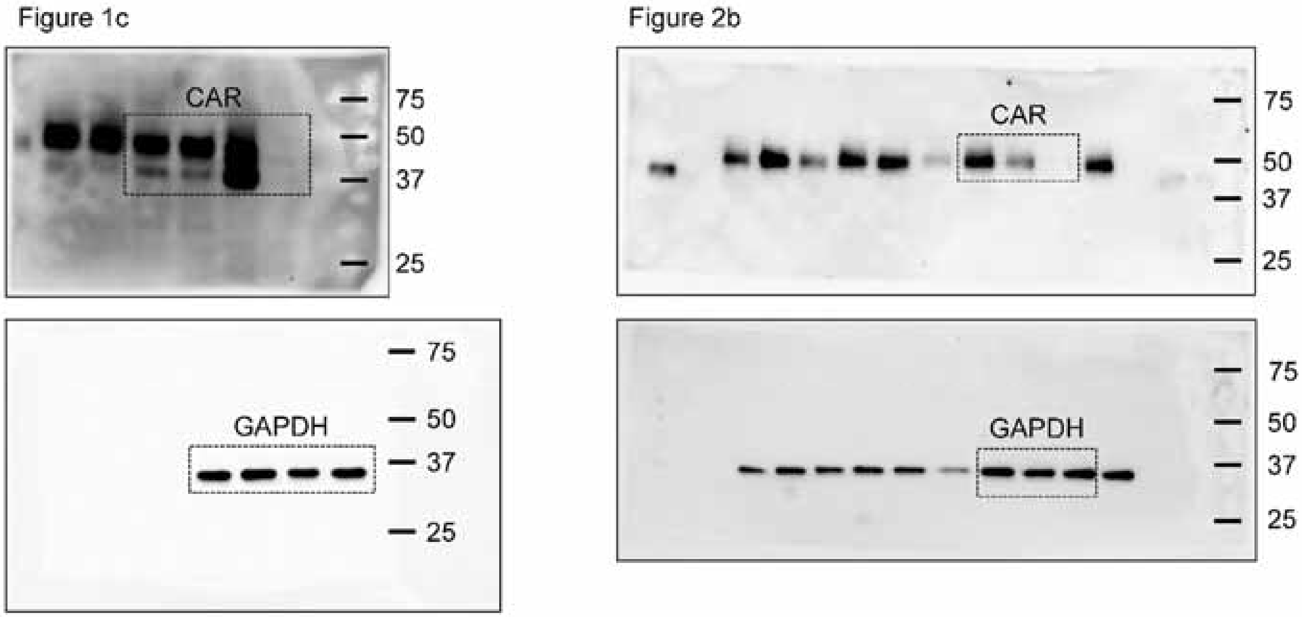

Supplementary Fig. 4. Western Blot uncropped images

Gating strategy of hepatocytes: RELATED TO FIGURE 1d

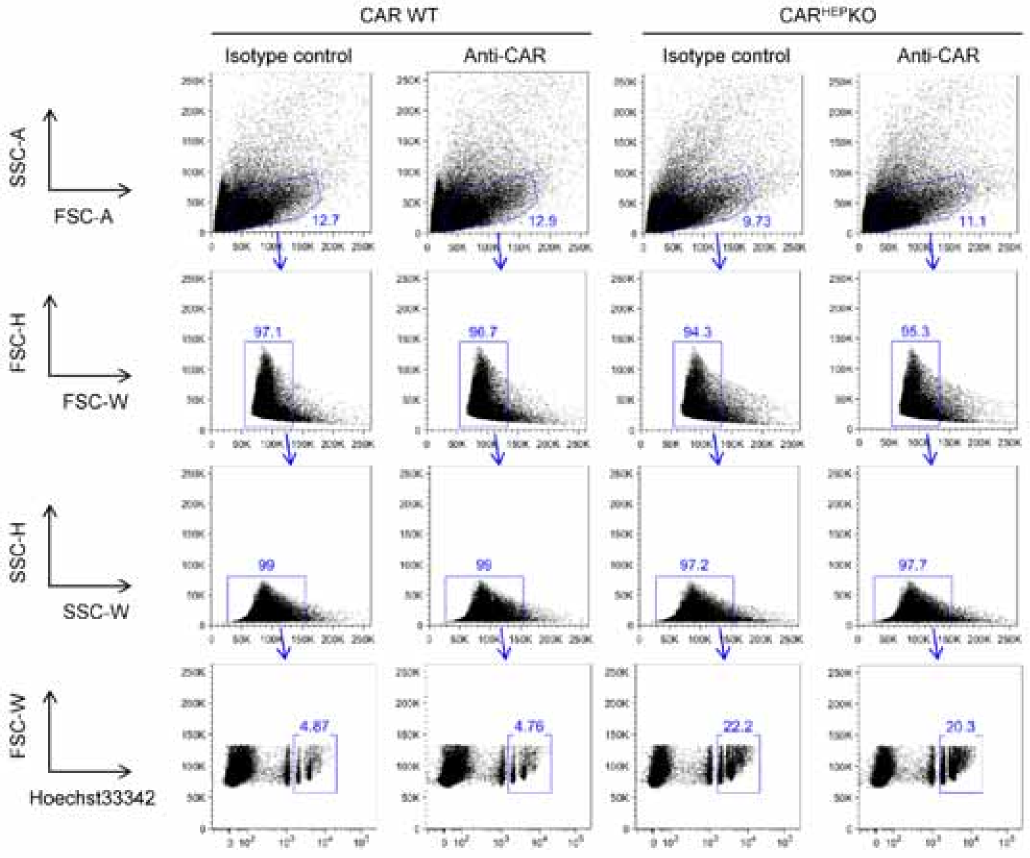

Supplementary Fig. 5. Flow cytometry gating strategies for hepatocytes

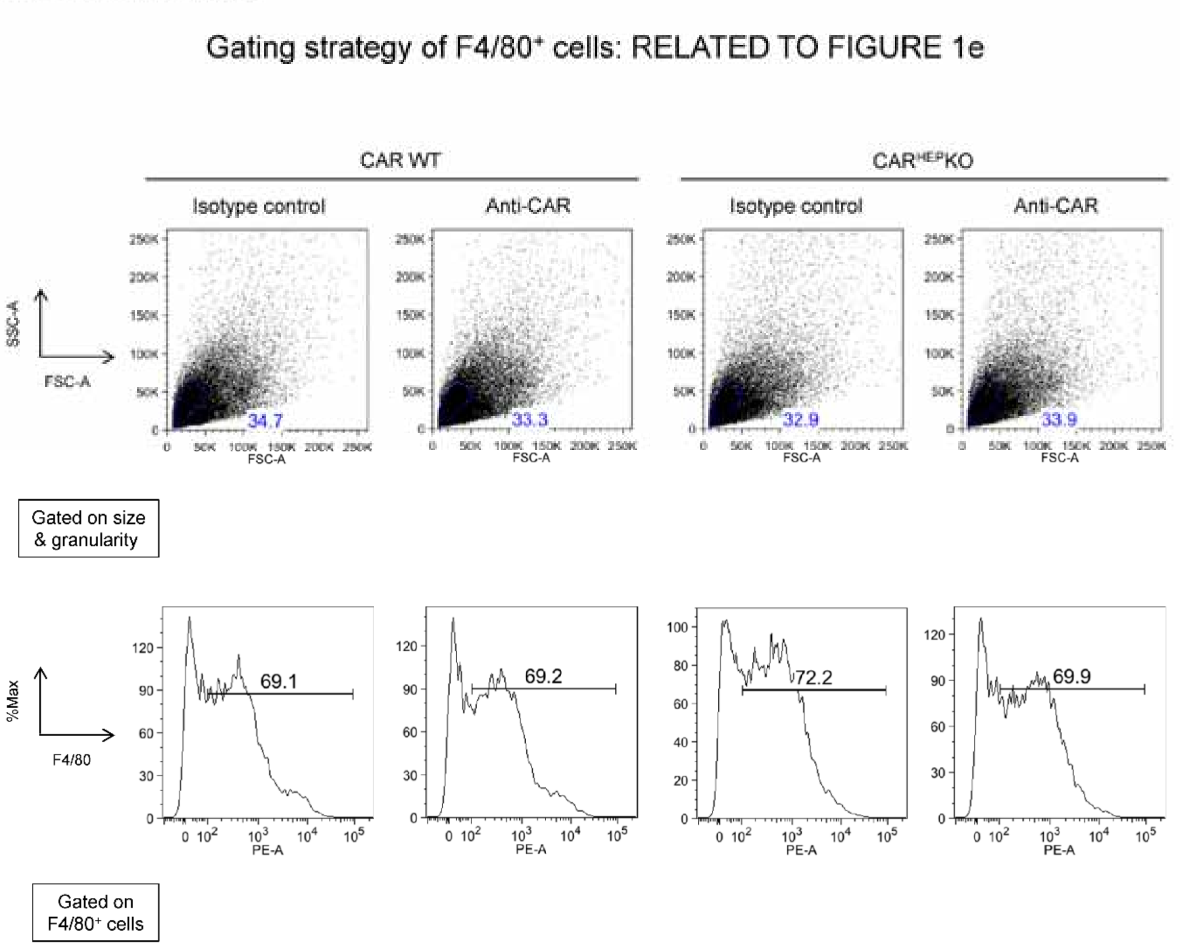

Supplementary Fig. 5 (ctd): Flow cytometry gating strategies for Kupffer cells

GATING STRATEGY FOR pDC experiments: RELATED TO FIGURE 5

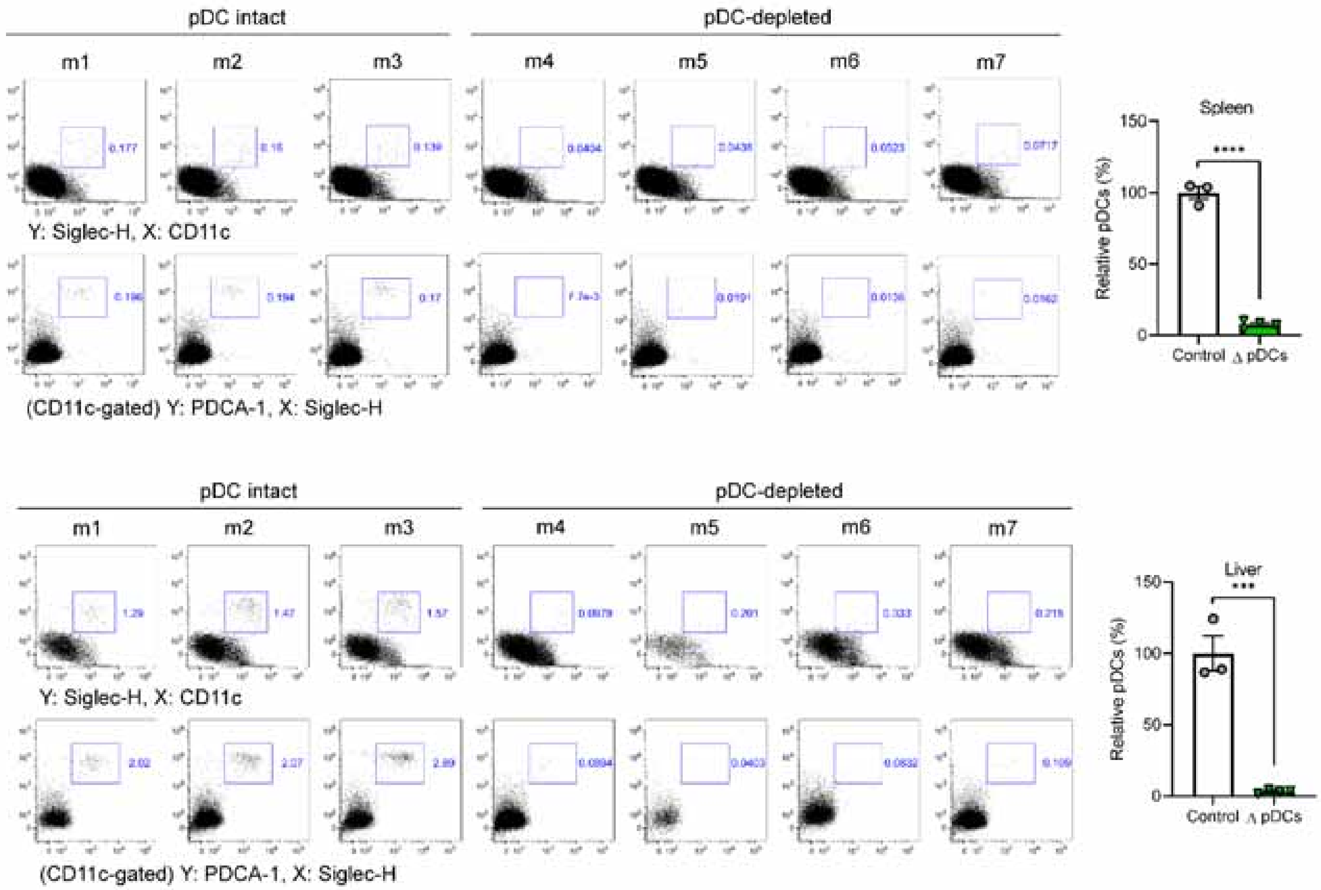

Supplementary Fig. 5 (ctd): Flow cytometry gating strategies for pDCs
