# Supplementary material for: Hepatocytes trap and silence coxsackieviruses, protecting against systemic disease in mice
Source: Commun Biol. 2020 Oct 16;3:580. doi: 10.1038/s42003-020-01303-7 (PMC7568585; doi:10.1038/s42003-020-01303-7)
Supplement: Supplementary file 2 — Description of Additional Supplementary Items [file 42003_2020_1303_MOESM2_ESM.pdf]

Description of Additional Supplementary Items

Supplementary Movie 1. B6 (wild type) mice were infected with CVB3. 24 hours later, the mice were sacrificed, and a vibratome section of liver was stained with phalloidin (red, to detect F-actin), and with an antibody specific for the viral VP1 protein (green). Nuclei were stained with Hoechst (blue). The VP1 signals (closely apposed to F-actin, thus appearing yellow) are located in sinusoids, not in hepatocytes.

Supplementary Movie 2. IRF1KO mice were infected with CVB3. 24 hours later, the mice were sacrificed, and a vibratome section of liver was stained with phalloidin (red, to detect F-actin), and with an antibody specific for the viral VP1 protein (green). Nuclei were stained with Hoechst (blue). VP1 signal is readily observed in occasional hepatocytes.

Supplementary Data 1. Source data for the graphs in the main figures.
