# Supplementary material for: Hepatocytes trap and silence coxsackieviruses, protecting against systemic disease in mice
Source: Commun Biol. 2020 Oct 16;3:580. doi: 10.1038/s42003-020-01303-7 (PMC7568585; doi:10.1038/s42003-020-01303-7)
Supplement: Supplementary file 6 — Reporting Summary [file 42003_2020_1303_MOESM6_ESM.pdf]

nature research

Corresponding author(s): Taishi Kimura, JL Whitton

Last updated by author(s): Aug 31, 2020

Reporting Summary

Nature Research wishes to improve the reproducibility of the work that we publish. This form provides structure for consistency and transparency in reporting. For further information on Nature Research policies, see Authors & Referees and the Editorial Policy Checklist.

Statistics

For all statistical analyses, confirm that the following items are present in the figure legend, table legend, main text, or Methods section.

n/a	Confirmed	
☐	☒	The exact sample size (n) for each experimental group/condition, given as a discrete number and unit of measurement
☐	☒	A statement on whether measurements were taken from distinct samples or whether the same sample was measured repeatedly
☐	☒	The statistical test(s) used AND whether they are one- or two-sided<br>*Only common tests should be described solely by name; describe more complex techniques in the Methods section.*
☐	☒	A description of all covariates tested
☐	☒	A description of any assumptions or corrections, such as tests of normality and adjustment for multiple comparisons
☐	☒	A full description of the statistical parameters including central tendency (e.g. means) or other basic estimates (e.g. regression coefficient) AND variation (e.g. standard deviation) or associated estimates of uncertainty (e.g. confidence intervals)
☐	☒	For null hypothesis testing, the test statistic (e.g. F, t, r) with confidence intervals, effect sizes, degrees of freedom and P value noted<br>*Give P values as exact values whenever suitable.*
☒	☐	For Bayesian analysis, information on the choice of priors and Markov chain Monte Carlo settings
☒	☐	For hierarchical and complex designs, identification of the appropriate level for tests and full reporting of outcomes
☒	☐	Estimates of effect sizes (e.g. Cohen's d, Pearson's r), indicating how they were calculated

Our web collection on statistics for biologists contains articles on many of the points above.

Software and code

Policy information about availability of computer code

Data collection	Microsoft OneNote (Manual counting of the virus plaques), Zen (Confocal Microscopy analysis), the machine pre-installed software for ChemiDoc Imager (Western Blot and PCR array), the machine pre-installed software for Victor X3 (ELISA and ALT activity assay), FACS DIVA (Flow cytometry)
Data analysis	Microsoft Excel, GraphPad Prism, FlowJo, ImageJ, Imaris

For manuscripts utilizing custom algorithms or software that are central to the research but not yet described in published literature, software must be made available to editors/reviewers. We strongly encourage code deposition in a community repository (e.g. GitHub). See the Nature Research guidelines for submitting code & software for further information.

Data

Policy information about availability of data

All manuscripts must include a data availability statement. This statement should provide the following information, where applicable:

- Accession codes, unique identifiers, or web links for publicly available datasets
- A list of figures that have associated raw data
- A description of any restrictions on data availability

Relevant data and/or materials are in Extended Figures and available upon reasonable request from TK (tkimura@scripps.edu) and/or JLW (lwhitton@scripps.edu).

Field-specific reporting

Please select the one below that is the best fit for your research. If you are not sure, read the appropriate sections before making your selection.

☒ Life sciences ☐ Behavioural & social sciences ☐ Ecological, evolutionary & environmental sciences

For a reference copy of the document with all sections, see nature.com/documents/nr-reporting-summary-flat.pdf

Life sciences study design

All studies must disclose on these points even when the disclosure is negative.

Sample size	All experiments were designed to have at least three biological replicates (in vitro) and three individuals (in vivo). All in vivo experiments were performed at least two times independently, and the data are combined.
Data exclusions	No data were excluded.
Replication	All attempts at replication were successful.
Randomization	Randomization is not relevant to our in vitro study as viral infections of the cells examine the response of entire cell populations. Samples were not randomized for our in vivo study. Anayzed mice were age- and sex-matched and compared the phenotype between the examined groups.
Blinding	Investigators were not blinded during experiments.

Reporting for specific materials, systems and methods

We require information from authors about some types of materials, experimental systems and methods used in many studies. Here, indicate whether each material, system or method listed is relevant to your study. If you are not sure if a list item applies to your research, read the appropriate section before selecting a response.

Materials & experimental systems

n/a	Involved in the study	
☐	☒	Antibodies
☐	☒	Eukaryotic cell lines
☒	☐	Palaeontology
☐	☒	Animals and other organisms
☒	☐	Human research participants
☒	☐	Clinical data

Methods

n/a	Involved in the study	
☒	☐	ChIP-seq
☐	☒	Flow cytometry
☒	☐	MRI-based neuroimaging

Antibodies

Antibodies used	Antibodies against CD11c, PDCA-1 and Siglec-H were purchased from BioLegend. Antibody against CD16/32 was purchased from BD Sciences. anti-CXADR/CAR antibody was provided by Dr. Klingel, University Hospital Tübingen, Germany, and anti-GAPDH antibody (clone 6C5) was purchased from EMD Millipore. Mouse anti-Enterovirus VP1 (Clone 5-D8/1) was purchased from Mediagnost Germany. HRP-conjugated donkey anti-rabbit IgG and HRP-conjugated sheep anti-mouse IgG were purchased from GE Healthcare Life Sciences.
Validation	All antibodies were validated by manufacturer.

Eukaryotic cell lines

Policy information about cell lines

Cell line source(s)	HeLa cells, H2.35 cells, Primary hepatocytes (laboratory isolate), Primary hepatocytes (purchased from Lonza)
Authentication	HeLa cells, H2.35 cells and primary hepatocytes (purchased from Lonza) were authenticated by the manufacturer. Primary hepatocytes (laboratory isolate) were checked for hepatocyte-specific gene expression (Alb, Hnf4a and Afp) and no expression of irrelevant cell-specific gene (Myh6) by comparing to those expression in primary cardiomyocytes.
Mycoplasma contamination	HeLa cells, H2.35 cells and primary hepatocytes (purchased from Lonza) were tested negative for mycoplasma by the manufacturer. Primary hepatocytes (laboratory isolate) were not tested for mycoplasma.

Commonly misidentified lines (See ICLAC register)	N/A

Animals and other organisms

Policy information about studies involving animals; ARRIVE guidelines recommended for reporting animal research

Laboratory animals	CAR-floxed mice were a generous gift from Dr. Robert Ross at UC San Diego. CLEC4C-DTR mice (JAX 014176), Albumin-Cre transgenic mice (JAX 003574) and IRF1KO mice (JAX 002762) were purchased from the Jackson laboratory. C57BL/6 mice were purchased from the TSRI rodent breeding colony and the Jackson laboratory (JAX 000664).
Wild animals	N/A
Field-collected samples	N/A
Ethics oversight	N/A

Note that full information on the approval of the study protocol must also be provided in the manuscript.

Flow Cytometry

Plots

Confirm that:

☒ The axis labels state the marker and fluorochrome used (e.g. CD4-FITC).

☒ The axis scales are clearly visible. Include numbers along axes only for bottom left plot of group (a 'group' is an analysis of identical markers).

☒ All plots are contour plots with outliers or pseudocolor plots.

☒ A numerical value for number of cells or percentage (with statistics) is provided.

Methodology

Sample preparation	To determine the pDC population, mice were sacrificed and perfused with DPBS. Spleens and livers were isolated, cut into small pieces and incubated in Collagenase D solution [2% FBS, HG 1:100, 5M MgCl2/CaCl2 1:500, P/S/G 1:100, Collagenase D (Roche, #11088866001) 1mg/ml final, DNase 1:500 (20 µg/ml, Roche)] by shaking at 180 rpm at 37 °C for 30 min. Then, the tissues were mechanically disrupted. Splenic red blood cells were lysed with 0.83% NH4Cl. Hepatic immune cells were isolated using lympholite-M (Cedarlane, #CL5031) according to the manufacturer's instruction. After rising with PBS several times, followed by Fc-blocking with anti-CD16/32 (BD Biosciences #553142), immune cells were immunophenotyped with the fluorescently conjugated antibodies to cell surface markers.
Instrument	LSR-II
Software	FACS DIVA, FlowJo
Cell population abundance	Sorting was not performed in this study.
Gating strategy	All gating strategies are included in Extended Data Figure 5.

☒ Tick this box to confirm that a figure exemplifying the gating strategy is provided in the Supplementary Information.
